# Guidance for the management of venous thrombosis in unusual sites

**DOI:** 10.1007/s11239-015-1308-1

**Published:** 2016-01-16

**Authors:** Walter Ageno, Jan Beyer-Westendorf, David A. Garcia, Alejandro Lazo-Langner, Robert D. McBane, Maurizio Paciaroni

**Affiliations:** Department of Clinical and Experimental Medicine, University of Insubria, Via Guicciardini 9, 21100 Varese, Italy; Dresden University Hospital, Dresden, Germany; University of Washington, Seattle, WA USA; University of Western Ontario, London, ON Canada; Mayo College of Medicine, Rochester, MN USA; University of Perugia, Perugia, Italy

**Keywords:** Venous thromboembolism, Cerebral vein thrombosis, Splanchnic vein thrombosis, Retinal vein occlusion, Anticoagulants, Direct oral anticoagulants (DOAC), New oral anticoagulants (NOAC)

## Abstract

Venous thromboembolism (VTE) is a serious and often fatal medical condition with an increasing incidence. The treatment of VTE is undergoing tremendous changes with the introduction of the new direct oral anticoagulants and clinicians need to understand new treatment paradigms. This manuscript, initiated by the Anticoagulation Forum, provides clinical guidance based on existing guidelines and consensus expert opinion where guidelines are lacking. In this chapter, we address the management of patients presenting with venous thrombosis in unusual sites, such as cerebral vein thrombosis, splanchnic vein thrombosis, and retinal vein occlusion. These events are less common than venous thrombosis of the lower limbs or pulmonary embolism, but are often more challenging, both for the severity of clinical presentations and outcomes and for the substantial lack of adequate evidence from clinical trials. Based on the available data, we suggest anticoagulant treatment for all patients with cerebral vein thrombosis and splanchnic vein thrombosis. However, in both groups a non-negligible proportion of patients may present with concomitant bleeding at the time of diagnosis. This should not contraindicate immediate anticoagulation in patients with cerebral vein thrombosis, whereas for patients with splanchnic vein thrombosis anticoagulant treatment should be considered only after the bleeding source has been successfully treated and after a careful assessment of the risk of recurrence. Finally, there is no sufficient evidence to support the routine use of antithrombotic drugs in patients with retinal vein occlusion. Future studies need to assess the safety and efficacy of the direct oral anticoagulants in these settings.

## Introduction

Venous thromboembolism (VTE) can potentially occur in any section of the venous system. Although the most common clinical presentations involve the deep veins of the lower limbs and the pulmonary arteries, VTE is also diagnosed in the cerebral venous system, in the abdominal and pelvic veins, or in the retinal veins, among other sites. The occurrence of VTE in unusual sites represents a clinical challenge because of the potential severity of clinical outcomes and because treatment strategies are not supported by adequate evidence from clinical trials. In this guidance document, we will review available evidence on the management of cerebral vein thrombosis, splanchnic vein thrombosis, and retinal vein occlusion.

## Background

### Cerebral vein thrombosis

Cerebral vein thrombosis (CVT) most commonly affects young adults, with 75 % of events occurring in women, and has a wide spectrum of signs and symptoms, which may evolve suddenly or over the weeks [1]. Headache is the most frequent presenting symptom. Other clinical presentations include seizures, focal neurological deficits, altered consciousness, and papilledema, which can present in isolation or in association with other symptoms [2]. Risk factors associated with CVT include gender-related systemic prothrombotic factors such as the use of oral contraceptives or pregnancy and local risk factors such as head injury, neurological procedures, lumbar puncture, and infections, in particular otitis and mastoiditis, and meningitis [1].

The clinical outcome of CVT appears to be more favourable than with thrombosis of the cerebral arteries. Still, in a systematic review of the literature the estimated mortality rate was 5.6 % (range 0–15.2 %) during the acute phase of the disease and 9.4 % (0–39 %) after a follow up that ranged across studies from 6 months to 10.2 years [3]. Residual disability was detected in about 10.0 % of the patients after follow up [3]. Finally, the estimated annual incidence of recurrent venous thrombosis was reported to range between 2.0 and 2.4/100 patient years [4, 5].

### Splanchnic vein thrombosis

Splanchnic vein thrombosis (SVT) encompasses Budd–Chiari syndrome, portal vein thrombosis, mesenteric vein thrombosis, and splenic vein thrombosis. Of all symptoms, abdominal pain is the most frequent. Other clinical manifestations may be associated with the underlying disorder and/or may represent the consequence of the acute thrombosis, such as in the case of gastrointestinal bleeding and ascites [6]. Systemic risk factors such as hematologic disorders, autoimmune diseases and the use of hormonal therapy are the most common risk factors associated with Budd–Chiari syndrome, whereas local precipitating factors such as solid abdominal cancer, liver cirrhosis, intraabdominal inflammatory conditions, and surgery are the most common risk factors associated portal and mesenteric vein thrombosis [6]. Thus, a careful imaging of the abdominal organs often identifies underlying predisposing pathologies in these patients. Myeloproliferative neoplasms have emerged as a leading systemic cause of SVT, and screening for the JAK2V617F mutation should be considered in patients without a known major underlying provocative factor [7].

Overall survival after long-term follow up is lower than in patients with deep vein thrombosis of the lower limbs, and depends on the location of thrombosis and on underlying diseases [6]. Long-term sequelae include, among others, portal hypertension and liver cirrhosis [8]. Bleeding is commonly reported during follow up, and may be related to underlying diseases, esophageal varices and anticoagulant treatment [6, 9, 10]. The annual incidence of recurrent thrombosis was reported to be about 2.5/100 patient years [9, 11].

### Retinal vein occlusion

Retinal vein occlusion (RVO) is the second most common retinal vascular disease after diabetic retinopathy. It may affect the central retinal vein or its branches, and the most common clinical presentation is sudden, unilateral and painless loss of vision, generally caused by macular edema [12]. Branch RVO may present with peripheral visual-field defect only or may be asymptomatic [12]. Both central and branch RVO can present with or without signs of ischemic lesions. Similarly to CVT and SVT, predisposing factors can be local or systemic. Local risk factors include open-angle glaucoma and inflammatory conditions, while cardiovascular risk factors such as hypertension, diabetes, and hyperlipidemia are the most common systemic risk factors associated with RVO [13]. However, the mechanism of action of these systemic predisposing factors may relate to damage of the adjacent artery.

Visual prognosis is related to initial visual acuity, and it is better for branch RVO than for central RVO, and for non-ischemic RVO than for ischemic RVO [14, 15]. In addition to permanent visual loss, other late complications include vitreous haemorrhages, retinal detachment or neovascular glaucoma [13]. The risk of recurrence is not negligible and recurrence may occur in the same eye or in the fellow eye [16].

Given the lack of high-quality evidence in this area, the anticoagulation Forum has developed the current document in order to suggest options for clinicians managing patients with venous thromboembolism in unusual sites. The issues addressed throughout include: indication for anticoagulation, selection of antithrombotic agents, contraindications to treatment, role of thrombolysis.

## Methods

To provide guidance on the therapeutic management of unusual site venous thrombosis, we first developed a number of pivotal practical questions for each site of thrombosis considered in this document (Table 1). Questions were developed by consensus from the authors.

**Table 1:** Guidance questions to be considered

*Cerebral vein thrombosis*
(1) Should anticoagulant drugs in patients with CVT be considered?
(2) Is concomitant bleeding a contraindication to the use of anticoagulant treatment?
(3) Are low molecular weight heparin (LMWH) and unfractionated heparin (UFH) similarly effective and safe for the acute phase treatment?
(4) Is the standard treatment regimen used for patients with deep vein thrombosis of the lower limbs (i.e. heparins for approximately 5–7 days and warfarin possibly started on the first treatment day) applicable to all patients?
(5) Is there a role for thrombolysis?
(6) What is the optimal duration of anticoagulant therapy after a first episode of CVT?
(7) Is there a role for the direct oral anticoagulants?
*Splanchnic vein thrombosis*
(1) Should all patients with SVT receive anticoagulant treatment?
(2) Is gastrointestinal bleeding at the time of diagnosis a contraindication to anticoagulant therapy?
(3) Is the standard treatment regimen used for patients with deep vein thrombosis of the lower limbs (i.e. heparins for approximately 5–7 days and warfarin possibly started on the first treatment day) applicable to all treatable patients?
(4) What factors should be considered before starting anticoagulant treatment in a patient with liver cirrhosis?
(5) Is there a role for thrombolysis?
(6) What are the factors driving treatment duration?
(7) Is there a role for the direct oral anticoagulants?
*Retinal vein occlusion*
(1) Should antithrombotic drugs in patients with newly diagnosed RVO be considered?
(2) Which antithrombotic treatment should be preferred?
(3) Is there a role for thrombolysis?
(4) How long should antithrombotic drugs be administered in RVO patients?
(5) Is there a rationale for prescribing long-term aspirin treatment?

To address these questions, a literature search of MEDLINE and EMBASE from January 2004 to August 2014 was conducted. The following search terms were used and combined: anticoagulant treatment, anticoagulant therapy, antithrombotic treatment, heparin, low molecular weight heparin, enoxaparin, nadroparin, dalteparin, certoparin, bemiparin, tinzaparin, parnaparin, reviparin, vitamin K antagonists, warfarin, acenocoumarol, phenprocoumon, thrombolysis, thrombolytic treatment, fibrinolytic agent, fibrinolysis, urokinase, tenecteplase, alteplase, rtPA, tPA; aspirin, ticlopidine, clopidogrel; cerebral vein thrombosis, cerebral venous thrombosis, cerebral veins, sinus thrombosis intracranial; splanchnic vein thrombosis, splanchnic venous thrombosis, splanchnic veins, portal vein thrombosis, mesenteric vein thrombosis, splenic vein thrombosis, hepatic vein thrombosis, Budd–Chiari syndrome; retinal vein occlusion, retinal vein thrombosis. The search strategy was restricted to papers published in English. Detailed information on the results of the literature search are available upon request.

For papers published before 2004, we only considered the most important studies that were likely to influence our responses to the questions. These studies were selected and suggested by the authors of this guidance document.

Because we anticipated that in this setting very few randomized controlled (RCT) trials would have been available, our selection was not restricted to a specific study design, but included RCTs, prospective and retrospective cohort studies, and case series describing a minimum of ten cases.

## Guidance

### Cerebral vein thrombosis

1. Should anticoagulant drugs in patients with CVT be considered?

Two RCTs and one meta-analysis have compared UFH and LMWH (nadroparin) with placebo in the acute treatment of CVT [17–19]. The meta-analysis of the two clinical trials found a reduction in death or dependency with the use of UFH or LMWH [relative risk (RR) 0.33; 95 % CI 0.08–1.21 for death and 0.46; 95 % CI 0.16–1.31 for dependency], but this was not statistically significant, likely because of the very small number of patients enrolled in the studies (79 patients). There were two episodes of pulmonary embolism (one was fatal) in the placebo group. The use of anticoagulant treatment was not associated with an increased risk of symptomatic intracranial haemorrhage. The latest American Heart Association/American Stroke Association, European Federation of Neurological Societies, and American College of Chest Physicians guidelines recommend that anticoagulation should be given to all patients with CVT who do not have contraindications [20–22].

(2) Is concomitant bleeding a contraindication to the use of anticoagulant treatment?

Concomitant intracerebral bleeding is not uncommon at the time of CVT diagnosis [1] and is mainly related to increased intracranial pressure secondary to thrombosis. The safety of anticoagulant therapy in patients with concomitant intracranial bleeding has never been specifically assessed in clinical studies, but available data suggest that intracerebral bleeding should not represent a contraindication to anticoagulation. Specifically, Einhaupl et al. [17] reported that three patients with previous intracerebral haemorrhage recovered completely and had no recurrent haemorrhages in the UFH group, and DeBruijn et al. [18] reported no recurrent intracerebral haemorrhages or clinical worsening in the 15 patients who presented with intracranial bleeding in the LMWH group. In the ISCVT prospective cohort study, about 40 % of patients treated with LMWH or UFH had intracranial haemorrhage at presentation [23].

#### **Guidance Statement**

*Concomitant intracerebral bleeding at the time of CVT diagnosis should not contraindicate the use of anticoagulant treatment. Anticoagulants with a shorter half-life (UFH or LMWH) should be administered over the first days of therapy and the introduction of oral anticoagulants should be postponed until the patient is clinically stable and the neuro-radiological picture improves*.

(3) Are low molecular weight heparin (LMWH) and unfractionated heparin (UFH) similarly effective and safe for the acute phase treatment?

No direct comparisons between UFH and LMWH are available, but data from an indirect comparison based on the results of the ISCVT study suggest that LMWH may be more effective and safer than UFH [23]. In particular, patients treated with LMWH in this prospective cohort study were more likely to be functionally independent after 6 months after adjustment for prognostic factors and imbalances and less likely to have new intracerebral haemorrhages, whereas no difference in mortality rates was observed [23]. Of patients with intracranial haemorrhage at baseline, 12 % experienced a new bleeding during follow up in the group treated with LMWH and 28 % in the group treated with UFH [23]. The European Federation of Neurological Societies guideline recommends LMWH over UFH because of the practical advantages and also based upon the results of RCTs carried out in patients with deep vein thrombosis of the lower limbs [20]. However, in a recent international survey on treatment strategies for CVT patients, 64 % of physicians reported using UFH and 36 % LMWH [24]. The advantages of UFH include its shorter half-life and its potential reversibility, which become crucial for the treatment of clinically unstable patients or for those requiring invasive procedures such as lumbar punctures or decompressive hemicraniectomy [25, 26]. If LMWH is used, a twice daily regimen may be preferred over a once daily regimen because of the lower peaks and higher troughs associated with the twice daily administration.

#### **Guidance Statement**

*LMWH and UFH appear to be similarly effective and safe for the acute phase treatment. The use of LMWH may be preferred over UFH for the majority of patients due to the practical advantages. The short half-life of UFH may be preferred over LMWH for clinically unstable patients or for patients requiring invasive procedures*.

(4) Is the standard treatment regimen used for patients with deep vein thrombosis of the lower limbs (i.e. heparins for approximately 5–7 days and warfarin possibly started on the first treatment day) applicable to all patients?

No clinical studies have compared different durations of parenteral treatment in patients with CVT. However, it would be reasonable to delay oral anticoagulant initiation because of the potential need for invasive procedures, the possible us of thrombolysis in case of clinical worsening, or the potential risk of new intracranial bleeding over the first days of therapy [27].

#### **Guidance Statement**

*The introduction of oral anticoagulants should be considered when the patient is clinically stable; that is, in the presence of normalized level of consciousness or a remission of mental confusion, improvement in headache and focal neurological deficits and improvement in the neuro-radiological picture*.

(5) Is there a role for thrombolysis?

One Cochrane review and three systematic reviews assessed the role of thrombolytic drugs in the acute treatment of CVT [28–31]. There are no RCTs that have evaluated thrombolytic therapy in this setting, and the results of the available studies, when combined, suggest a non-negligible risk of bleeding complications while the efficacy of the treatment is not assessable.

The European Federation of Neurological Societies guidelines suggest thrombolysis for patients whose conditions deteriorate despite adequate anticoagulant therapy and in whom other causes of deterioration have been ruled out, possibly in the absence of large intracranial haemorrhages and threatening herniation [20]. Intra-vascular thrombolysis may be preferred if adequate expertise is available, but no data on direct comparison with systemic thrombolysis exist.

#### **Guidance Statement**

*The use of either systemic or local thrombolytic therapy should be restricted to very selected high-risk patients only, such as in patients who deteriorate despite adequate anticoagulant therapy in whom other causes of deterioration have been ruled-out*.

(6) What is the optimal duration of anticoagulant therapy after a first episode of CVT and what are the main factors driving treatment duration?

No clinical studies have specifically addressed the issue of the optimal duration of secondary prevention of venous thromboembolism with anticoagulant therapies in patients with CVT. The results of a systematic review of the literature report a recurrence rate of 2.8 % after pooling the results of 13 studies with follow-up duration ranging between 12 and 145 months [3]. In addition, the rate of VTE occurring in other sites (apart from CVT) was 3.7 % [3]. Two more recent studies reported an annual recurrence rate of any VTE of 2.0 % [4] and of 3.5 % [5], respectively, after discontinuation of anticoagulant treatment and a median follow up of 6 years and 40 months, respectively. Male sex and severe thrombophilia were independently associated with the risk of recurrence in the first study [4]; previous VTE was the only independent predictor of recurrence in the second study [5]. In the ISCVT study, male gender and polycythemia or thrombocythemia were independently associated with the risk of recurrence [32]. This study had a shorter median follow up of 13 months.

The European Federation of Neurology Societies guidelines suggest 3 months of anticoagulant treatment when CVT is secondary to a transient risk factor, 6–12 months when CVT is unprovoked and in the presence of mild thrombophilia, and indefinite treatment duration when CVT is recurrent or associated with severe thrombophilia [20].

#### **Guidance Statement**

*Anticoagulant treatment for a minimum of 3 months should be considered in patients with transient risk factors. Patients without known risk factors should be considered for 6–12 months of anticoagulation. It appears safe to discontinue anticoagulant treatment in the presence of transient risk factors such as oral contraceptive use, while indefinite treatment duration should be considered for patients with recurrent CVT and in patients with permanent major risk factors including severe thrombophilia (antithrombin, protein C or protein S deficiency; antiphospholipid antibodies syndrome; homozygous factor V Leiden or prothrombin gene mutation or combined heterozygous mutation)*.

(7) Is there a role for the direct oral anticoagulants?

No CVT patients have been enrolled in phase III clinical trials of the direct oral anticoagulants. We found only one report of seven patients with CVT treated with rivaroxaban [33]. In this report, no major bleeding events were documented and the overall outcome was reported to be favourable. Given the results of the RCTs comparing the direct oral anticoagulants with standard treatment in patients with deep vein thrombosis and/or pulmonary embolism and given the fast onset and offset of action of these compounds, it seems plausible that the direct oral anticoagulants will have a role also in this setting. However, additional evidence is needed before recommending for or against their use in this setting.

#### **Guidance Statement**

*Given the absence of clinical experience with the use of the direct oral anticoagulants in this setting, there is no evidence for or against their use in clinical practice until additional data from clinical studies will become available. If a decision to use these agents is made, their use should be considered off-label and careful patient counselling and clinical monitoring should follow. Ideally, patients receiving direct oral anticoagulants should be included in prospective cohort studies aimed to fill this knowledge gap*.

### Splanchnic vein thrombosis

1. Should all patients with SVT receive anticoagulant treatment?

We found no RCTs assessing anticoagulant treatment in this setting and available evidence is based on the results of observational studies. The results of these studies suggest that the use of anticoagulants is associated with improved survival [6], with a lower risk of recurrence [9, 10, 34], and with improved recanalization [33, 34], but also with an increased risk of gastrointestinal bleeding [6].

An increasing number of SVTs are diagnosed incidentally in asymptomatic patients. Whether these events are associated with a lower risk of recurrence as compared to symptomatic events, and whether anticoagulation is effective in these patients is unknown. It should be noted that the majority of patients with incidentally detected SVT have major permanent risk factors such as cancer or liver cirrhosis [35, 36].

The American College of Chest Physicians guidelines recommend anticoagulation for symptomatic SVT patients, and no anticoagulation for asymptomatic patients with incidentally detected events [37]. Anticoagulation for patients with acute and chronic portal vein thrombosis and Budd Chiari syndrome is recommended by the American Association for the Study of Liver Diseases [38].

However, patients with SVT may present with active gastrointestinal bleeding or with a very high risk of bleeding due to concomitant underlying disorders. In these patients, the risks associated with anticoagulation may offset its benefits. In a prospective study aimed at describing treatment strategies for SVT patients in real life, more than 20 % of patients did not receive anticoagulant treatment [39]. Factors associated with no treatment included gastrointestinal bleeding at presentation, thrombocytopenia, cancer, hepatic cirrhosis, and incidental diagnosis of SVT [39].

#### **Guidance Statement**

*Anticoagulant treatment should be considered for all patients with symptomatic SVT and no evidence of active bleeding. The decision to administer anticoagulants to patients with incidentally detected, asymptomatic SVT should be made on an individual basis, carefully balancing the presence of risk factors for recurrence (e.g. underlying prothrombotic conditions) and the risk of bleeding*.

(2) Is gastrointestinal bleeding at the time of diagnosis a contraindication to anticoagulant therapy?

Gastrointestinal bleeding may be present at the time of SVT diagnosis in up to 25 % of patients [8]. It is in most cases associated with esophageal varices, but it can also occur after intestinal infarction in patients with mesenteric vein thrombosis. Active gastrointestinal bleeding represents a contraindication to anticoagulant treatment, but anticoagulation should be considered in patients with previous bleeding, in particular when at high risk of thrombus extension or recurrence. However, the optimal timing for starting anticoagulant therapy is unknown and it should be decided on an individual basis taking into account the management of bleeding sources and the presence of additional risk factors for bleeding. For example, in some cases, it may make sense to start anticoagulation only after the high pressure due to venous obstruction has been relieved.

#### **Guidance Statement**

*In the presence of active bleeding, anticoagulant treatment should be initiated only when the bleeding source has been successfully treated and the patient is clinically stable. The decision to start anticoagulant treatment should be driven by the presence of major risk factors for recurrence, the ability to address the underlying cause for bleeding, and by the location and extent of thrombosis*.

(3) Is the standard treatment regimen used for patients with deep vein thrombosis of the lower limbs (i.e. heparins for approximately 5–7 days and warfarin possibly started on the first treatment day) applicable to all treatable patients?

SVT is associated with solid cancer in approximately 22–27 % of patients [6, 39]. LMWH has been shown to be more effective than warfarin in patients with cancer-associated deep vein thrombosis of the lower limbs or pulmonary embolism and, for this reason, LWMH is the current treatment of choice at least for the first 3–6 months in this population [37]. Although no studies comparing LMWH with warfarin are available in SVT patients, it is plausible that the clinical benefit of LMWH is similar also in this setting

The initiation of warfarin on the first days of treatment may not be practical or safe in high bleeding risk patients such as patients with known esophageal varices or thrombocytopenia, or in unstable patients who may be requiring invasive procedures such as portosystemic shunting. In these patients, treatment with LMWH alone (or with UFH in some circumstances) should be preferred, due to its shorter half-life. Dose reductions of LMWH should be considered in patients with thrombocytopenia, although there is no adequate evidence to support any specific dosing algorithm based on platelet count. Some experts suggest to use a half therapeutic dose (i.e. 100 IU/kg daily) in patients with a platelet count between 50,000 and 100,000 mm^3^, and a prophylactic dose in patients with a platelet count between 30,000 and 50,000 mm^3^, and no anticoagulation below 30,000 mm^3^. Other experts suggest the dose be reduced only when the platelet count is below 50,000 mm^3^ in patients without concomitant major risk factors for bleeding (e.g. esophageal varices). For patients with hepatic injury, prolonged hospitalization with poor nutrition, or antibiotic therapy, initially warfarin dosing should be judicious so as not to overshoot the INR target [2.0–3.0].

#### **Guidance Statement**

*This treatment regimen may not be appropriate for patients with cancer-associated SVT or for patients with major risk factors for bleeding (e.g. liver cirrhosis and/or known esophageal varices, thrombocytopenia), for whom an initial course of treatment with LMWH (3–6 months for cancer patients) is preferable. In patients with thrombocytopenia, reduced doses of LMWH should be used (prophylactic or half therapeutic dose) according to the platelet count and to the concomitant presence of additional risk factors for bleeding. For all other patients, the introduction of warfarin should be considered only when the patient is clinically stable. In patients at very high risk of bleeding or possibly requiring invasive procedures the use of UFH may be preferred over LMWH*.

(4) What factors should be considered before starting anticoagulant treatment in a patient with liver cirrhosis?

Approximately 24–28 % of SVT patients have known liver cirrhosis [6, 39]. In these patients, bleeding risk associated with the presence of portal hypertension needs to be carefully assessed. Esophageal varices have been consistently reported to be associated with an increased risk of bleeding in SVT patients [6, 9], but their presence does not represent an absolute contraindication to anticoagulant therapy, because treatment of SVT may improve the portal hypertension. However, before starting anticoagulants, routine endoscopic screening of esophageal varices and prophylactic treatment of variceal bleeding, if indicated, may be warranted. In a management study by Senzolo et al., 35 patients with portal vein thrombosis and cirrhosis underwent endoscopic screening of esophageal varices, and patients with previous variceal bleeding and those with grade II esophageal varices with red signs and grade III varices were banded [40]. Anticoagulation with LMWH was started not earlier than 15 days after the last banding session. One episode of variceal bleeding was reported in this study. In another study, 55 cirrhotic patients with portal vein thrombosis were treated with either warfarin or LMWH and the main study outcome was the rate of complete recanalization of the portal vein [41]. Of interest, initiation of anticoagulant treatment within the first 2 weeks after diagnosis was the only predictive factor for complete recanalization. The majority of these patients (78 %) received beta-blockers for prophylaxis of variceal bleeding.

#### **Guidance Statement**

*Routine endoscopic screening of esophageal varices and prophylactic treatment of variceal bleeding should be considered for all cirrhotic patients with SVT. In patients who are not actively bleeding, anticoagulant treatment should be started as soon as possible with initially reduced doses of LMWH (either prophylactic doses or half therapeutic doses also according to the platelet count). Full doses of LMWH should be started only after the completion of the banding treatment*.

(5) Is there a role for thrombolysis?

Case reports describe the use of thrombolytic therapy in SVT patients [42–47]. Different strategies were used, including systemic intravenous administration or catheterization of the superior mesenteric artery or the portal vein. In some of these cases this approach was reported to be beneficial, but others reported a high risk of major or even fatal bleeding [47]. For this reason, the use of thrombolytic agents should be limited to very selected cases, such as mesenteric vein thrombosis patients with signs of intestinal ischemia or patients whose conditions deteriorate despite adequate anticoagulant therapy. There are insufficient data to suggest a preference for local or systemic lysis, and the choice should be based on local experience and preferences.

#### **Guidance Statement**

*The use of thrombolytic agents should be limited to very selected cases, such as patients with mesenteric vein thrombosis and with signs of intestinal ischemia or patients whose conditions deteriorate despite adequate anticoagulant therapy*.

(6) What is the optimal duration of anticoagulant therapy after a first episode of SVT and what are the factors driving treatment duration?

In a large retrospective cohort of 832 patients with portal, mesenteric, splenic, and hepatic vein thrombosis, the annual incidence of recurrent VTE after a mean follow-up of 27 months was 3.5 per 100 patient-years [6]. In this study, only 28 % of patients received warfarin, which in 75 % of cases was prescribed for an indefinite period of time. Hormonal therapy was the only independent risk factor for recurrence, while the use of warfarin was not protective. In a retrospective study enrolling 136 non-cirrhotic patients with portal vein thrombosis only, the incidence rate of thrombotic events after a median follow-up of 46 months was 5.5 per 100 patient-years [9]. Fifty-two patients were not treated with anticoagulants, 30 were treated for a definite period of time (mean treatment duration not reported), and 54 remained on anticoagulant treatment during the whole study period. The presence of an underlying prothrombotic state was independently associated with the risk of recurrence, while the use of anticoagulant therapy was associated with a statistically significant reduction in recurrent events. In the retrospective cohort study by Spaander et al., 66 of 120 non-cirrhotic patients with portal vein thrombosis received anticoagulant treatment, and the overall thrombotic risk at 1, 5 and 10 years was 4, 8, and 27 %, respectively [10]. The use of anticoagulant therapy was associated with a lower risk of recurrence, but also with a significant increase in bleeding risk. Recurrent thrombosis, but not bleeding, was associated with poor survival. Finally, the annual risk of recurrence in 77 patients with mesenteric vein thrombosis all treated with vitamin K antagonists was found to be 4.6 % person-years in the about 40 % of patients who discontinued anticoagulant treatment [11].

The American Association for the Study of Liver Diseases recommends anticoagulant therapy for at least 3 months for all patients with acute portal vein thrombosis, and long-term anticoagulation for patients with concomitant mesenteric vein thrombosis or patients with permanent thrombotic risk factors [38]. The majority of SVT patients have underlying prothrombotic risk factors, which in most cases are permanent. Recurrent thrombosis may be severe since in about one fourth of cases it occurs as hepatic, mesenteric, or splenic infarctions [9]. Anticoagulant treatment is effective in preventing recurrence, but bleeding rates reported in SVT patients appear to be higher than those reported in patients with deep vein thrombosis of the lower limbs. However, in the study on patients with MVT only, case-fatality rate of thrombosis was significantly higher than that of gastrointestinal bleeding [11]. Whether patients with SVT plus a symptomatic myeloproliferative syndrome with positive JAK2 V617F mutation can safely transition from anticoagulation to aspirin therapy once they have started cytoreductive therapy or JAK2 inhibitors (e.g. with hydroxyurea or interferon) is not known.

#### **Guidance Statement**

*Anticoagulant treatment should be administered for a minimum of 3 months to all SVT patients. It appears safe to discontinue anticoagulant treatment in the presence of major transient risk factors, such as surgery or infections. For all other patients, including patients with cirrhosis, cancer including myeloproliferative neoplasms, or autoimmune disorders, indefinite treatment duration should be considered with periodic careful assessment of the risks and benefits*.

(7) Is there a role for the direct oral anticoagulants?

No SVT patients have been enrolled in phase III clinical trials of the direct oral anticoagulants. We found only two published case reports of PVT or MVT patients treated with rivaroxaban [48, 49]. Although direct oral anticoagulants represent important alternatives to LMWH and warfarin also in this setting, the reported increased risk of gastrointestinal bleeding in phase III clinical trials, at least with some molecules, remains a matter of concern. Furthermore, the direct oral anticoagulants are contraindicated in patients with acute or chronic severe liver impairment as a result of their partial metabolism through the CYP 3A4 system [50]. Thus, additional evidence is needed; pending such evidence, we can neither recommend for or against the use of direct oral anticoagulants in the management of SVT patients.

#### **Guidance Statement**

*Given the absence of clinical experience with the use of the direct oral anticoagulants in this setting, there is no evidence for or against their use in the management of patients with SVT. If a decision to use these agents is made, their use should be considered off-label and careful patient counselling and clinical monitoring should follow. Ideally, patients receiving direct oral anticoagulants should be included in prospective cohort studies aimed to fill this knowledge gap*.

### Retinal vein occlusion

1. Should antithrombotic drugs in patients with newly diagnosed RVO be considered?

A systematic review of the literature identified only one RCT comparing an antithrombotic drug with placebo in 89 patients [51, 52]. In this study, the use of ticlopidine administered for 6 months was associated with a trend toward improved visual acuity [52]. Since then, no other studies have used placebo as a comparator. In a prospective cohort study of 686 RVO patients, patients treated with aspirin had a worse visual outcome than patients who did not receive aspirin [53]. The guidelines of the Royal College of Ophthalmologists recommend against the use of antithrombotic drugs for patients with RVO [54]. However, because of the thrombotic nature of the occlusion and because of the association between RVO and cardiovascular risk factors, aspirin is commonly prescribed to RVO patients, at least in some institutions [53, 55].

#### **Guidance Statement**

*There is no high-quality evidence to support routine use of antithrombotic drugs for RVO patients. Neither the benefits nor the risks of antithrombotic therapy have been well defined in this clinical setting. That notwithstanding, antithrombotic treatment may be considered in selected patients with recent onset of symptoms and no local risk factors for thrombosis (e.g. glaucoma) or in patients with underlying major prothrombotic risk factors such as the antiphospholipid antibodies syndrome*.

(2) Which antithrombotic treatment should be preferred?

Three small RCTs have compared the efficacy and safety of LMWH and aspirin for the treatment of patients with RVO [56–58]. A meta-analysis of these studies found a statistically significant improvement in visual acuity and a 78 % risk reduction in adverse ocular outcomes with the use of LMWH, with no increased risk of vitreous haemorrhage [59]. In these studies, treatment was started within 15 [56] or 30 days [57, 58] after the onset of symptoms and was continued for 20–30 days [57, 58] or 3 months [56]. The use of anticoagulants during the acute phase of disease remains controversial and largely varies among different institutions. Some experts support the use of anticoagulation in patients with concomitant severe thrombophilia, such as the antiphospholipid antibody syndrome.

#### **Guidance Statement**

*If antithrombotic therapy is used, LMWH administered at therapeutic doses may be considered for the acute phase treatment of RVO*.

(3) Is there a role for thrombolysis?

Two RCTs have compared fibrinolytic therapy with no treatment [60] or hemodilution [61]. In the first study patients received intravenous streptokinase for 72 h followed by UFH and then warfarin for 6 months [60]. Treatment was started within 7 days from symptoms onset. In the second study, patients received intravenous rt-PA for 60 min, UFH for 8 days and aspirin for 12 weeks and treatment was started within 11 days from symptoms onset [61]. There was a trend toward improved visual acuity in the groups of patients receiving active treatments, with similar rates of vitreous haemorrhage.

#### **Guidance Statement**

*The use of locally administered thrombolytic therapy should be limited to very selected cases, such as RVO patients with total visual loss*.

(4) How long should antithrombotic drugs be administered in RVO patients?

Few studies reported on the long-term incidence of RVO recurrence. One study reported an annual incidence of ipsilateral RVO recurrence of about 1 % [62], in another study the incidence of recurrence in the same eye was 0.9 % after 2 years and 2.5 % after 4 years [63]. In this latter study that enrolled 1,108 patients, the cumulative probability of recurrence in the fellow eye was 7.7 % after 2 years and 11.9 % after 4 years [63]. Pooling the results of 24 studies on patients with branch RVO and 53 studies with central RVO, a systematic review of the literature reported that 5 % of patients with central RVO developed a recurrent RVO over a 1-year period [16]. The role for antithrombotic agents in the long-term secondary prevention of RVO remains unexplored and there are no available data to suggest that antithrombotic treatment reduces the risk of recurrence. Treatment duration in RCTs ranged from few weeks to 6 months.

#### **Guidance Statement**

*If antithrombotic therapy is used, LMWH should be administered for a period of 1–3 months. Aspirin should be administered for an indefinite period of time, when indicated*.

(5) Is there a rationale for prescribing long-term aspirin treatment?

A number of studies have reported an association between RVO and hypertension or diabetes [64–67]. Other studies have reported higher mortality rates from cardiovascular disease in patients with RVO than in patients with no RVO [68, 69]. Combining the populations of two large prospective cohort studies with a total of 8,384 individuals, Cugati et al. reported that subjects aged less than 70 years with RVO had a significantly higher risk of cardiovascular mortality than patients without RVO (HR 2.5, 95 % CI 1.2–5.2) [69]. This increased risk was not observed in older patients after adjusting for cardiovascular risk factors. Finally, in a systematic review and meta-analysis, Khan and colleagues have recently assessed the 10-year Framingham risk for patients with RVO [70]. The estimated 10-year Framingham risk score in subjects with RVO was 10.1 % (95 % CI 9.9–10.2), and it was significantly higher than the average risk score of 6 % calculated in the general population.

Whether RVO may occur as the first clinical manifestation of arteriosclerosis in some patients, and whether long-term treatment with aspirin may effectively prevent cardiovascular morbidity and mortality in these patients is unknown.

#### **Guidance Statement**

*The decision to prescribe long-term aspirin treatment should be based on an individual patient assessment and should also take into account other concomitant indications for the primary or secondary prevention of cardiovascular disease*.

## Conclusion

The treatment of venous thrombosis occurring in unusual sites is particularly challenging because of the lack of evidence from clinical trials. The prescription of standard therapeutic regimens that are usually recommended for patients with deep vein thrombosis of the lower limbs or pulmonary embolism needs to be carefully assessed on an individual basis, because the optimal timing of introduction, the optimal duration, and the dosages of anticoagulant drugs may need to be adapted according to the clinical presentation and to the presence of underlying disorders. For example, in patients with CVT concomitant intracerebral bleeding is frequently encountered and requires the prolonged use of anticoagulants with a short half-life (UFH or LMWH) until the clinical stability is achieved and the neuro-radiological picture shows clear improvement. Conversely, in patients with SVT presenting with concomitant gastrointestinal bleeding the use of any anticoagulant can only be considered when the bleeding source is treated and the patient is stable. Not uncommonly, SVT patients do not receive anticoagulation because the risk of bleeding is perceived to persistently outweight the risk of recurrence. Table 2 provides a summary of therapeutic interventions for VTE in unusual sites. CVT most commonly occurs in young females with gender specific risk factors and has a favorable clinical history with a relatively low long-term risk of recurrence. For this reason, indefinite anticoagulation is only suggested for patients with recurrent disease or in the uncommon presence of permanent risk factors. SVT is frequently associated with major permanent risk factors such as liver cirrhosis or cancer, which place patients at a high long-term risk of recurrence. Thus, for the majority of patients, with the exclusion of those with SVT secondary to surgery or an acute infection, indefinite treatment duration is suggested. RVO appears to be a different disease entity caused by either local risk factors or systemic cardiovascular risk factors. For this reason, the need for anticoagulant therapy is extremely uncertain and antiplatelet agents are frequently prescribed in clinical practice, despite the lack of sufficient evidence to support the role of any antithrombotic drug. Table 3 contains a summary of guidance statements for the management of VTE in unusual sites.

**Table 2:** Summary of therapeutic interventions for VTE in unusual sites

	Antithrombotic drug of choice	Suggested dosing	Suggested duration	Search for underlying conditions	Role of thrombolysis
Cerebral vein thrombosis					
Without active bleeding	LMWH (UFH), VKA recommended for most pts. (DOAC^a^)	LMWH: 200 aXa/kg/day; UFH: 2.5-fold PTT VKA: INR 2–3; DOAC^a^: approved VTE treatment dosages	At least 3 months for all pts. Discontinuation in pts. with transient risk factors Long-term in pts. with permanent major risk factors including severe thrombophilia^b^	Thrombophilia estrogen exposure PNH, JAK-2 mutation, myeloproliferative neoplasm Steroid use/abuse	Selected patients with severe symptoms (coma) or deterioration during anticoagulant therapy
With active bleeding	UFH or LMWH; VKA (DOAC^a^) only after stabilization	LMWH: 100–200 aXa/kg/day; UFH: 2–2.5-fold PTT VKA: INR 2–3; DOAC^a^: approved VTE treatment dosages			
Splanchnic vein thrombosis					
Without active bleeding	LMWH (UFH) recommended for all pts. with symptomatic SVT In asymptomatic (incidental) SVT, treatment decision need to balance risk of thromboembolic and bleeding complications (DOAC^a^)	LMWH: 200 aXa/kg/day; UFH: 2.5-fold PTT VKA: INR 2–3 DOAC^a^: approved VTE treatment dosages	At least 3 months for all pts. Discontinuation in pts. with transient risk factors (surgery, infections) Long-term in pts. in all other pts (including cirrhosis, solid cancer, myeloproliferative neoplasms, severe thrombophilia (antithrombin, protein C or protein S deficiency; APS; homozygous factor V Leiden or prothrombin gene mutation or combined heterozygous mutation; PNH)	In cirrhotic patients limited search for underlying conditions, but screening for esophageal or fundus varices (and prophylactic treatment) In non-cirrhotic patients search for solid cancers, PNH (if suggested by concomitant signs of hemolysis), JAK-2 mutation, myeloproliferative neoplasms Autoimmune disease Intraabdominal infections Thrombophilia Steroid use/abuse	Selected cases: Patients with mesenteric vein thrombosis and intestinal ischemia Deterioration despite adequate anticoagulant therapy
With active bleeding	Treatment of bleeding before initiation of any antithrombotic therapy	No antithrombotic treatment in acute bleeding, but start immediately after successful treatment of bleeding site			No thrombolysis in actively bleeding patients or patients at high bleeding risk
Retinal vein	No routine use of antithrombotic drugs but anticoagulation may be considered in selected patients with recent onset of symptoms and no local risk factors for thrombosis	Only if indicated: LMWH: 200 aXa/kg/day Patient with relevant arterial disease should be treated with aspirin (100 mg/day) instead	LMWH should be administered for a period of 1–3 months, aspirin should be administered for an indefinite period of time, when indicated	Search for arterial disease assess and treat cardiovascular risk factors	Locally administered thrombolytic therapy should be limited to very selected cases, such as RVO patients with total visual loss

^a^DOAC should only be used with great caution due to lack of experience. Patient informed consent and reasons against VKA use should be documented

^b^Severe thrombophilia: antithrombin, protein C or protein S deficiency; APS; homozygous factor V Leiden or prothrombin gene mutation or combined heterozygous mutation; PNH

**Table 3:** Summary of guidance statements

Question	Guidance statement
*Cerebral vein thrombosis*	
(1) Should anticoagulant drugs in patients with CVT be considered?	Anticoagulant drugs should be considered for all patients with CVT
(2) Is concomitant bleeding a contraindication to the use of anticoagulant treatment?	Concomitant intracerebral bleeding at the time of CVT diagnosis should not contraindicate the use of anticoagulant treatment. Anticoagulants with a shorter half-life (UFH or LMWH) should be administered over the first days of therapy and the introduction of warfarin should be postponed until the patient is clinically stable and the neuro-radiological picture improves
(3) Are low molecular weight heparin (LMWH) and unfractionated heparin (UFH) similarly effective and safe for the acute phase treatment?	LMWH and UFH appear to be similarly effective and safe for the acute phase treatment. The use of LMWH may be preferred over UFH for the majority of patients due to the practical advantages. The short half-life of UFH may be preferred over LMWH for clinically unstable patients or for patients requiring invasive procedures
(4) Is the standard treatment regimen used for patients with deep vein thrombosis of the lower limbs (i.e. heparins for approximately 5–7 days and warfarin possibly started on the first treatment day) applicable to all patients?	The introduction of warfarin should be considered when the patient is clinically stable; that is, in the presence of normalized level of consciousness or a remission of mental confusion, improvement in headache and focal neurological deficits and improvement in the neuro-radiological picture
(5) Is there a role for thrombolysis?	The use of either systemic or local thrombolytic therapy should be restricted to very selected high-risk patients only, such as in patients who deteriorate despite adequate anticoagulant therapy in whom other causes of deterioration have been ruled-out
(6) What is the optimal duration of anticoagulant therapy after a first episode of CVT?	Anticoagulant treatment for a minimum of 3 months should be considered in patients with transient risk factors. Patients without known risk factors should be considered for 6–12 months of anticoagulation. It appears safe to discontinue anticoagulant treatment in the presence of transient risk factors such as oral contraceptive use, while indefinite treatment duration should be considered for patients with recurrent CVT and in patients with permanent major risk factors including severe thrombophilia (antithrombin, protein C or protein S deficiency; antiphospholipid antibodies syndrome; homozygous factor V Leiden or prothrombin gene mutation or combined heterozygous mutation)
(7) Is there a role for the direct oral anticoagulants?	Given the absence of clinical experience with the use of the direct oral anticoagulants in this setting, there is no evidence for or against their use in clinical practice until additional data from clinical studies will become available. If a decision to use these agents is made, their use should be considered off-label and careful patient counselling and clinical monitoring should follow. Ideally, patients receiving direct oral anticoagulants should be included in prospective cohort studies aimed to fill this knowledge gap
*Splanchnic vein thrombosis*	
(1) Should all patients with SVT receive anticoagulant treatment?	Anticoagulant treatment should be considered for all patients with symptomatic SVT and no evidence of active bleeding. The decision to administer anticoagulants to patients with incidentally detected, asymptomatic SVT should be made on an individual basis, carefully balancing the presence of risk factors for recurrence (e.g. underlying prothrombotic conditions) and the risk of bleeding
(2) Is gastrointestinal bleeding at the time of diagnosis a contraindication to anticoagulant therapy?	In the presence of active bleeding, anticoagulant treatment should be initiated only when the bleeding source has been successfully treated and the patient is clinically stable. The decision to start anticoagulant treatment should be driven by the presence of major risk factors for recurrence, the ability to address the underlying cause for bleeding, and by the location and extent of thrombosis
(3) Is the standard treatment regimen used for patients with deep vein thrombosis of the lower limbs (i.e. heparins for approximately 5–7 days and warfarin possibly started on the first treatment day) applicable to all treatable patients?	This treatment regimen may not be appropriate for patients with cancer-associated SVT or for patients with major risk factors for bleeding (e.g. liver cirrhosis and/or known esophageal varices, thrombocytopenia), for whom an initial course of treatment with LMWH (3–6 months for cancer patients) is preferable. In patients with thrombocytopenia, reduced doses of LMWH should be used (prophylactic or half therapeutic dose) according to the platelet count and to the concomitant presence of additional risk factors for bleeding. For all other patients, the introduction of warfarin should be considered only when the patient is clinically stable. In patients at very high risk of bleeding or possibly requiring invasive procedures the use of UFH may be preferred over LMWH
(4) What factors should be considered before starting anticoagulant treatment in a patient with liver cirrhosis?	Routine endoscopic screening of esophageal varices and prophylactic treatment of variceal bleeding should be considered for all cirrhotic patients with SVT. In patients who are not actively bleeding, anticoagulant treatment should be started as soon as possible with initially reduced doses of LMWH (either prophylactic doses or half therapeutic doses also according to the platelet count). Full doses of LMWH should be started only after the completion of the banding treatment
(5) Is there a role for thrombolysis?	The use of thrombolytic agents should be limited to very selected cases, such as patients with mesenteric vein thrombosis and with signs of intestinal ischemia or patients whose conditions deteriorate despite adequate anticoagulant therapy
(6) What is the optimal duration of anticoagulant therapy after a first episode of SVT?	Anticoagulant treatment should be administered for a minimum of 3 months to all SVT patients. It appears safe to discontinue anticoagulant treatment in the presence of major transient risk factors, such as surgery or infections. For all other patients, including patients with cirrhosis, cancer including myeloproliferative neoplasms, or autoimmune disorders, indefinite treatment duration should be considered with periodic careful assessment of the risks and benefits
(7) Is there a role for the direct oral anticoagulants?	Given the absence of clinical experience with the use of the direct oral anticoagulants in this setting, there is no evidence for or against their use in the management of patients with SVT. If a decision to use these agents is made, their use should be considered off-label and careful patient counselling and clinical monitoring should follow. Ideally, patients receiving direct oral anticoagulants should be included in prospective cohort studies aimed to fill this knowledge gap
*Retinal vein occlusion*	
(1) Should antithrombotic drugs in patients with newly diagnosed RVO be considered?	There is no high-quality evidence to support routine use of antithrombotic drugs for RVO patients. Neither the benefits nor the risks of antithrombotic therapy have been well defined in this clinical setting. That notwithstanding, antithrombotic treatment may be considered in selected patients with recent onset of symptoms and no local risk factors for thrombosis (e.g. glaucoma) or in patients with underlying major prothrombotic risk factors such as the antiphospholipid antibodies syndrome
(2) Which antithrombotic treatment should be preferred?	If antithrombotic therapy is used, LMWH administered at therapeutic doses may be considered for the acute phase treatment of RVO
(3) Is there a role for thrombolysis?	The use of locally administered thrombolytic therapy should be limited to very selected cases, such as RVO patients with total visual loss
(4) How long should antithrombotic drugs be administered in RVO patients?	If antithrombotic therapy is used, LMWH should be administered for a period of 1–3 months. Aspirin should be administered for an indefinite period of time, when indicated
(5) Is there a rationale for prescribing long-term aspirin treatment?	The decision to prescribe long-term aspirin treatment should be based on an individual patient assessment and should also take into account other concomitant indications for the primary or secondary prevention of cardiovascular disease
